# Attraction of the sand fly *Nyssomyia neivai* (Diptera: Psychodidae) to chemical compounds in a wind tunnel

**DOI:** 10.1186/s13071-015-0748-y

**Published:** 2015-03-07

**Authors:** Vicente Estevam Machado, Arlene Gonçalves Corrêa, Thais Marchi Goulart, Flávia Benini da Rocha Silva, Dennys Ghenry Samillan Ortiz, Mara Cristina Pinto

**Affiliations:** Departamento de Ciências Biológicas, Faculdade de Ciências Farmacêuticas, Universidade Estadual Júlio de Mesquita Filho, UNESP, 14801-902 Araraquara, SP Brazil; Departamento de Química, Universidade Federal de São Carlos, 13565-905 São Carlos, SP Brazil; Departamento de Zoologia Animal, Universidade Estadual de Campinas, 13083-970 Campinas, SP Brazil; Universidade de Franca, 14404-611 Franca, SP Brazil

**Keywords:** Sand flies, Wind tunnel, Attractiveness, Alcohols, Plant volatiles, Olfactometry, *Nyssomyia neivai*, Octanol, Hexanol

## Abstract

**Background:**

Similar to other hematophagous insects, male and female sand flies must feed on plants to obtain sugar and, subsequently, energy to complete their life cycles. A large number of compounds emitted by plants may act as volatile signals to these insects. Primary alcohols have been detected in some plants, but in small amounts. In a previous report, the attractiveness of saturated primary alcohols with 7 to 9 carbons was evaluated for *Lutzomyia longipalpis*, the vector of American visceral leishmaniasis, with positive results.

**Methods:**

In the present study, a wide range of primary alcohols, 3 to 10 carbons, were tested to investigate their attractiveness to another sand fly species, *Nyssomyia neivai*, a putative vector of American cutaneous leishmaniasis. The mixture of compounds that induced the best sand fly response was also evaluated.

**Results:**

Of the eight compounds evaluated, hexanol and octanol elicited the best attractive responses for sand fly females.

**Conclusion:**

Phytochemicals may be an interesting source of search for new sand fly attractants.

## Background

Generally, the concept of kairomones for hematophagous insects is related to host volatiles, and the development of new lures is focused on those compounds. However, considering that hematophagous insects also feed on plant tissues, phytochemical attractants are very interesting targets, but less studied compared with kairomones from vertebrate animals [1].

There have been behavioural reports on the attractiveness of floral odours or single floral compounds to mosquitoes [2-4]. For sand flies, studies with plants [5-7] fruits [8] and honey [9] have shown their effective roles as attractants, but there has been no isolation of compounds from these different sources.

In a previous study conducted in a wind tunnel, the attractiveness of the host kairomone 1-octen-3-ol was compared with three saturated primary alcohols (1-heptanol, 1-octanol and 1-nonanol) for the sand fly *Lutzomyia longipalpis* [10]. Those saturated primary alcohols are present in small quantities in some plant volatiles. The results demonstrated a relative attractive response of female *L. longipalpis* to 1-heptanol and 1-nonanol. Such results indicate that other saturated primary alcohols may also be attractive to sand fly species. The present study investigated the attractiveness of a broad range of saturated primary alcohols (3 to 10 carbons) for *Nyssomyia neivai*, a putative vector of cutaneous leishmaniasis in South America [11]. The compounds that evoked the best responses were combined and evaluated for their attractiveness.

## Methods

### Sand flies and laboratory maintenance

Insects were collected, using manual aspiration on the wall of one house located at the edge of the Mogi-Guaçu River, northeast center of São Paulo State, Brazil (21 ° 35′13’S 48 ° 04′15’W). In the laboratory, the insects were maintained in netting cages with a 30% solution of sucrose fed *ad libitum* under controlled conditions (26 ± 1°C, 80–90%, 12:12 (L:D) photoperiod).

### Experiments in the wind tunnel

The experiments were performed in a wind tunnel (previously described) [12] within three to four days after the field collection of insects. Tests were performed from 9:00 to 19:00 and, at the end of the assay, the insects were identified according to the classification proposed by Galati [13].

Groups of three females were placed in a releasing chamber, for a total of 30 different insects for each concentration of the evaluated compounds. The releasing chambers were placed in the wind tunnel 50 cm from the stimulus. Tests lasted for 2 min, and two behaviours were evaluated: activation and attraction. Activation was defined as the number of sand flies that left the releasing chamber, and attraction was defined as the number of sand flies that reached the stimulus.

The compounds evaluated were: 1-propanol (99.5%), 1-butanol (99.4%), 1-pentanol (>99.5%), 1-hexanol (98%), 1-heptanol (98%), 1-octanol (>99%), 1- nonanol (98%) and 1-decanol (>99%) (hereafter propanol, butanol, pentanol, hexanol, heptanol, octanol, nonanol and decanol) (Sigma-Aldrich). The compound 1-octen3-ol (hereafter octenol) has been already shown to be attractive to *N. neivai* [12], so it was used as a positive control. All compounds were diluted in hexane and tested at concentrations of 10%, 50% and 100% (neat). When necessary, a lower concentration (5%) was also used. Each compound was delivered (200 μL) on a filter paper (4 × 4 cm) in the entrance of the tunnel. The control was hexane presented the same way prior to testing the other compounds.

After these tests, the three compounds with the best responses (heptanol, octanol, and nonanol) were combined (1:1:1) and subsequently evaluated in the wind tunnel.

The interval between a compound and another was at least two hours, when the tunnel was cleaned with hexane and allowed to dry.

### Statistical analysis

Chi square tests were used to evaluate the different proportions of sand fly females activated and attracted by each compound. Initially, the tests were conducted for all concentrations simultaneously. If a significant difference was verified, each of the two groups was compared separately. The statistical analyses were performed using BioEstat(available in http://www.mamiraua.org.br/pt-br/downloads/programas/bioestat-versao-53/).

## Results

Considering the attraction behaviour, sand flies responded in a clear dose-dependent manner to pentanol, hexanol, octanol and octenol. Propanol and butanol were not significantly different from the control for activation (propanol X^2^ = 6.9, df = 3, P = 0.07; butanol X^2^ = 6.7 df = 3, P = 0.1) or attraction (propanol X^2^ = 1.1, df = 3, P = 0.8; butanol X^2^ = 7.6, df = 3, P = 0.1). The other evaluated compounds presented some degree of activation (pentanol, X^2^ = 32.9, df = 3, P <0.0001; hexanol X^2^ = 68.7, df = 3, P <0.0001; heptanol X^2^ = 47.7, df = 3, P <0.0001; octanol X^2^ = 60.6 df = 3, P <0 0.0001; nonanol X^2^ = 46.3, df = 3, P < 0.0001; decanol X^2^ = 21.7, df = 3, P <0.0001) or attraction (pentanol, X^2^ = 41.6, df = 3, P < 0.0001; hexanol X^2^ = 50.6, df = 3, P <0.0001; heptanol X^2^ = 13.8, df = 3, P = 0.008; octanol X^2^ = 37.9 df = 3, P < 0.0001; nonanol X^2^ = 22.7, df = 3, P < 0.0001; decanol X^2^ = 11.3, df = 3, P = 0.01) for females *N. neivai* sand flies (Table 1).

**Table 1:** **Percentages of activated and attracted** ***N. neivai*** **females to individual alcohols and mixture of three alcohols (n = 30 for each compound)**

**Compound**	**Activated (%)**	**Attracted (%)**
Control	13 a	7 a
Propanol 10%	7 a	7 a
Propanol 50%	7 a	3 a
Propanol 100%	27 a	10 a
Control	13 a	7 a
Butanol 10%	27 a	10 a
Butanol 50%	30 a	13 a
Butanol 100%	43 a	30 a
Control	13 a	7 a
Pentanol 10%	40 ab	10 a
Pentanol 50%	**70 bc**	63 b
Pentanol 100%	**80 c**	67 b
Control	13 a	7 a
Hexanol 10%	30 a	17 a
Hexanol 50%	**90 b**	57 b
Hexanol100%	**100 b**	**87 c**
Control	13 a	7 a
Heptanol 5%	53 b	47 b
Heptanol 10%	**83 b**	43 b
Heptanol 50%	**87bc**	40 b
Heptanol 100%	**77 b**	33 a
Control	13 a	7 a
Octanol 5%	**77 b**	57 b
Octanol 10%	**80 b**	**70 b**
Octanol 50%	**83 b**	**70 b**
Octanol 100%	**97 b**	**73 b**
Control	13 a	7 a
Nonanol 5%	**70 b**	53 b
Nonanol 10%	**87 b**	43 b
Nonanol 50%	**83 b**	63 b
Nonanol 100%	**70 b**	47 b
Control	13 a	7 a
Decanol 10%	40 a	20 a
Decanol 50%	**70 b**	37 b
Decanol 100%	57 b	40 b
Control	13 a	7 a
Octenol 10%	13 a	10 a
Octenol 50%	**73 b**	47 b
Octenol 100%	**80 b**	**80 bc**
Mixture*	**97**	63

For each compound, different letters within a column indicate significant differences (P < 0.05). Bold signs indicates values ≥ 70%; a cut off for responses based on octenol. *Mixture = heptanol, octanol and nonanol (1:1:1).

With octenol as a positive control and 70% used as a cut off for responses, pentanol, hexanol, heptanol, octanol, nonanol and, to a lesser extent, decanol, elicited activation behaviours. However, according to the same criteria, hexanol and octanol were the best attractants for sand fly females. Octanol was even better than octenol at lower concentrations (50% and 10%).

The concentration of 5% was added to trials with heptanol, octanol and nonanol because they elicited high responses of activation at 10%. Those compounds were mixed (1:1:1) and tested again. The results showed that 93% of females were activated and 63% were attracted by the mixture. There was an improvement in activation that was not reflected for attraction.

## Discussion

Our study compared the attractiveness of saturated primary alcohols to sand flies with 1-octen-3-ol, an unsaturated secondary alcohol, used as lure for hematophagous insects. The results emphasised that, similar to a previous study with the sand fly species *L. longipalpis* [10], such compounds also act as activators and/or attractants for *N. neivai*. The results are also in agreement with our prior survey [12] and showed that flies taken directly from the field can be used in laboratory olfactometer experiments, in spite of different physiological conditions, because they showed consistently higher activation and attraction responses to the evaluated compounds than to air control.

Octenol is a known host kairomone found in cattle [14], human breath [15] and human skin emanation [16]. For sand flies, octenol has been shown to elicit a response in *L. longipalpis* in electrophysiological recordings [17] and attractive behaviours in a wind tunnel [10]. For *Nyssomyia intermedia* and *N. neivai*, octenol also elicited attractive responses in field studies [18,19]. In addition to its occurrence in mammals, octenol is also identified in many studies with volatile organic compounds (VOC) in different species of plants such as watermelon [20], flowers of lucerne [21] and mango [22].

The alcohols used in the present study are not host kairomones, but they are present in plant volatiles. For example, octanol and hexanol are present in watermelon [20] and strawberry [23]; and butanol, pentanol, hexanol, and octanol are present in guava [24]. Considering that sand flies require sugar meals from plants to survive, those compounds could be considered to be phytochemical attractants.

Previous reports have demonstrated the attractiveness of plants to sand flies [7,8,25]. A laboratory study in a wind tunnel evaluated unifloral honey odours as attractants for different populations of three sand flies species, and the responses were species and population specific [9].

Despite the evidence of plant attractiveness to sand flies, there is a gap in the identification of phytochemical attractants isolated from the plants. The main advantage of phytochemical attractants for hematophagous insects is that they lure males and females of all ages and females in all gonotrophic states [1]. Our results show that, in addition to host kairomones, other compounds, such as phytochemical, should also be investigated as attractants for sand flies.

## Conclusions

The compounds hexanol and octanol elicited the best attractive responses for *N. neivai* females in bioassays. The next step is to evaluate if those compounds will be attractive for other sand fly species in field.
